# Ferroptosis is important for *Toxoplasma gondii* replication and virulence *in vitro* and *in vivo*

**DOI:** 10.1080/21505594.2025.2530164

**Published:** 2025-07-16

**Authors:** Ling-Yu Li, Chun-Xue Zhou, Bing Han, Hany M. Elsheikha, Hui-Jie Qiu, Xu-Dian An, Ting Zeng, Dai-Ang Liu, Qing Yang, Xing-Quan Zhu, Huai-Yu Zhou

**Affiliations:** a Department of Pathogen Biology, School of Basic Medical Sciences, Cheeloo College of Medicine, Shandong University, Jinan, Shandong Province, PR China; b Faculty of Medicine and Health Sciences, School of Veterinary Medicine and Science, University of Nottingham, Loughborough, UK; c Laboratory of Parasitic Diseases, College of Veterinary Medicine, Shanxi Agricultural University, Jinzhong, Shanxi Province, China

**Keywords:** *T. gondii*, ferroptosis, GPX4, RSL3, mouse

## Abstract

The protozoan parasite *T. gondii* employs intricate mechanisms to exploit host cells while sustaining their viability, yet its interaction with ferroptosis – an iron-dependent cell death driven by lipid peroxidation – remains poorly defined. Here, we show *T. gondii* infection induces ferroptotic hallmarks in RAW264.7 macrophages, including elevated lactate dehydrogenase release, labile Fe^2 +^ accumulation, reactive oxygen species (ROS) generation, and lipid peroxidation. Molecular analyses revealed infection-induced downregulation of ferroptosis suppressor GPX4 and upregulation of pro-ferroptotic ACSL4 in macrophages and mice. Mechanistically, the SLC7A11/GPX4 axis governed parasite growth: knockdown of these genes promoted *T. gondii* replication, whereas overexpression restricted proliferation. Pharmacological studies showed ferroptosis inhibitor Fer-1 suppressed intracellular parasite proliferation. Notably, GPX4 inhibitor RSL3 exhibited context-dependent effects: pre-infection treatment enhanced replication, while post-infection administration inhibited growth. Direct RSL3 exposure induced time-dependent growth arrest in extracellular tachyzoites, associated with disrupted transcriptomes, increased lipid ROS, and downregulated parasite antioxidant genes (*TgPRX2*, *TgTPX1/2*, *TgNXN*), indicating redox homoeostasis impairment. In vivo murine studies corroborated this biphasic effect: therapeutic RSL3 administration post-infection significantly reduced parasite burdens across multiple organs (spleen, liver, kidney, brain) and improved survival rates, while prophylactic pretreatment exacerbated disease progression. We propose RSL3 exerts direct parasiticidal effects via oxidative damage but also enables early nutrient acquisition from ferroptosis-compromised host cells. These findings establish ferroptosis as a critical node in *T. gondii* pathogenesis, highlighting the parasite’s hijacking of host iron-lipid metabolism. The dual role of ferroptosis regulators underscores the host-pathogen metabolic complexity and positions the SLC7A11/GPX4 axis as a promising therapeutic target.

## Introduction

*T. gondii* is an opportunistic protozoan that causes diseases in immunocompromised individuals and can lead to adverse health consequence if acquired during pregnancy [1–3]. Current therapeutics cannot eliminate dormant stage of the parasites and are even toxic to the patient [4]. A vaccine against *T. gondii* infection would be an effective strategy to prevent infection. However, commercial vaccines against human toxoplasmosis have yet to be successfully developed [5]. There is a need to understand the interaction between *T. gondii* and the host to develop better therapeutic interventions. As an obligate intracellular parasite residing within a parasitophorous vacuole, *T. gondii* must acquire necessary nutrients, such as carbon sources, amino acids, lipids, trace elements, from the host cells [6]. Additionally, throughout the course of infection, *T. gondii* manipulates many cellular pathways to promote its own growth in the infected cells, often by modifying processes that control host cell fate, such as programmed cell death (PCD), to ensure parasite survival [7,8].

Ferroptosis is a type of PCD characterized by iron-dependent lipid peroxidation and has unique morphological and molecular attributes [9]. Ferroptosis can play a role in the pathogenesis of many infectious diseases because during infection increased production of reactive oxygen species (ROS) exceeds the capacity of antioxidant molecules such as glutathione (GSH), and therefore causes a disruption in the host redox homoeostasis [10–12]. For instance, Herpes simplex virus 1 (HSV-1) infection increases cell death and lipid peroxidation in U373 and HMC3 cells, and treatment with Fer-1 (a ferroptosis inhibitor) increases the survival of HSV-1-infected mice and alleviates HSV-1 encephalitis [13]. Inhibition of the solute carrier family 7 member 11 (SLC7A11), which is located on the plasma membrane, deprives cellular cysteine to induce GSH deletion [14]. Glutathione peroxidase 4 (GPX4) is an antioxidant enzyme that prevents lipid peroxidation in the presence of GSH [15]. Thus, inhibition of SLC7A11/GPX4 axis can increase cellular ferroptosis sensitivity. *Mycobacterium tuberculosis* (*Mtb*) infection in mice is associated with altered expression of SLC7A11 and GPX4, together with increased free iron, mitochondrial superoxide, and lipid peroxidation. And, *Mtb* infection in *Gpx4*-deficient mice results in marked increase in lung necrosis and bacterial burdens, whereas Fer-1-treated infected mice exhibit significant reduction in bacterial load [16,17].

Interestingly, characteristic features of ferroptosis are also featured in *T. gondii* infection, suggesting the possible contribution of ferroptosis to *T. gondii* pathogenesis. For example, *T. gondii* infection induces the production of ROS in human placental cells [18]. In murine placental trophoblasts infected by *T. gondii*, the level of ROS increases in a time-dependent manner, together with elevated malonaldehyde (MDA) and decreased level of GSH. Additionally, mitochondria cristae of the placental cells become fewer, discontinuous, or entirely disappear after *T. gondii* infection [19]. Ferroptosis occurs in hippocampus of mice after *T. gondii* infection, with increased iron augmentation and lipid peroxidation, and reduced GPX4 and GSH levels. Deferiprone (DFP) is an iron chelator, which can completely abrogate ferroptosis induced by the ferroptosis inducer RSL3 [20]. Mice treated with DFP have a lower *T. gondii* burden in the hippocampus and a prolonged survival time [21]. It was also noted that ferroptosis may have paradoxical anti-*T. gondii* effects, with RSL3 significantly inhibiting the growth of *T. gondii* in a dose-dependent manner [22]. Hence, it remains unclear whether ferroptosis restrains or promotes *T. gondii* infection. Better understanding of how *T. gondii* infection dysregulates or modifies ferroptosis may provide a potential therapeutic modality.

In this study, we investigate the impact of ferroptosis on *T. gondii* pathogenesis using in vitro and in vivo models. We demonstrate that *T. gondii* infection induces macrophage necrosis associated with labile iron accumulation, lipid peroxidation, and upregulation of ferroptosis-promoting proteins. Mechanistically, inhibition of the SLC7A11-GPX4 axis enhances parasite proliferation, whereas its activation restricts growth. Importantly, pre-infection treatment with RSL3 increases tissue parasite loads and reduces mouse survival, despite RSL3 exhibiting direct anti-parasitic activity in vitro. These findings reveal a dual role for ferroptosis in toxoplasmosis: while host cell ferroptosis may be exploited by the parasite for replication, direct pharmacological induction of ferroptosis can impair *T. gondii* survival. Our work underscores the complex interplay between ferroptosis and *T. gondii* infection, offering new insights into potential therapeutic targets for combating toxoplasmosis.

## Materials and methods

### Ethics statement

All animal experiments were approved by the Research Ethics Committee of Shandong University (ECSBMSSDU2019-2-006). The housing and care of experimental animals were carried out in strict accordance with the regulations of Good Animal Practice requirements of the Animal Ethics Procedures and Guidelines of the People’s Republic of China. All efforts were made to minimize the number of animals used in the study. *T. gondii* is a category 2 pathogen and infection by this pathogen represents an important global public health concern. Therefore, standard BSL2 practices and standard operating procedures were followed, and laboratory personnel involved in the study were advised about biohazards and ways to minimize the chances of exposure to infection. All experimental procedures were carried out in compliance with the ARRIVE guidelines.

### Cell culture and parasite strains

RAW264.7, human foreskin fibroblasts (HFFs) and HeLa cells were obtained from Shanghai Cell Bank, Chinese Academy of Sciences. These cells were cultured in Dulbecco’s modified Eagle’s medium (DMEM, VivaCell, C3113–0500, Shanghai, China) supplemented with 10% foetal bovine serum (FBS, VivaCell, C2910–0500, Shanghai, China) and 1% penicillin/streptomycin (NCM Biotech, C100C5, Suzhou, China). The cell culture was maintained at 37°C within a humidified incubator with a 5% CO_2_ atmosphere. To ensure cell line integrity, mycoplasma contamination was monitored every 6 months via PCR testing. Tachyzoites of *T. gondii* RH and ME49 strains were cultivated by serial passage in confluent monolayers of HFF cells in DMEM supplemented with 1% FBS at 37°C and 5% CO_2_.

### Animals

Female C57BL/6J mice (6–8 weeks old) were obtained from Jinan Pengyue Experimental Animal Breeding Co., Ltd. Mice were housed under pathogen-free conditions in a 12 h light/dark cycle with free access to food and water. Mice were acclimated for 1 week before being used in the experiment.

### Reagents and antibodies

The following chemicals were used: BODIPY™ 581/591 C11 (Invitrogen, D3861, Shanghai, China), FerroOrange (Dojindo, F374, Shanghai, China), RSL3 (Selleck, S8155, Shanghai, China), Ferrostatin-1 (Fer-1, Selleck, S7243, Shanghai, China), Deferoxamine mesylate (DFOM, Selleck, S5742, Shanghai, China), and EDTA (Solarbio, E1170, Beijing, China). For Western blotting, the following antibodies were used in this study: anti-ACSL4 antibody (Abcam, ab155282, Shanghai, China), anti-GPX4 antibody (Abcam, ab125066, Shanghai, China), anti-FSP1 antibody (Abcam, ab197896, Shanghai, China), anti-SLC7A11 antibody (Abcam, ab307601, Shanghai, China), anti-COX2 antibody (Abways, CY8852, Shanghai, China), anti-β-Tubulin antibody (Proteintech 10,094–1-AP, Wuhan, China), anti-GAPDH antibody (Proteintech 10,494–1-AP, Wuhan, China), and goat-anti-rabbit HRP (Proteintech, SA00001–2, Wuhan, China).

### RNAi and plasmid transfection

Human *SLC7A11*-siRNA and *GPX4*-siRNA were purchased from Sigma Aldrich (Shanghai). For transient silencing, duplexes of siRNA were transfected into HeLa cells using RFect siRNA/miRNA Transfection Reagent (Biodai 11,011, China) according to the manufacturer’s instruction. All plasmids were generated by cloning the corresponding cDNA (target) into the expression vector pCDNA3.1, and the primers used are listed in Table S1. All constructs were verified by DNA sequencing. The cells were transiently transfected with the plasmids pC-*SLC7A11* and pC-*GPX4* using Lipofectamine 2000 reagent (Invitrogen 11,668,019, USA) according to the manufacturer’s protocol. The expression efficiency was evaluated by Western blot analysis.

### LDH release assays

LDH release was measured using CytoTox 96® Non-Radioactive Cytotoxicity Assay (Promega, G1780, China) according to the manufacturer’s instructions. Briefly, RAW264.7 cells were seeded at a density of ~ 4 × 10^4^ cells/well in 96-well tissue culture plates. After infection by *T. gondii*(MOI 1 or 10)for 24 h, the supernatants were collected and incubated with LDH regents for 30 min in a sterile 96-well tissue culture plastic plate. The absorbance at 490 nm was measured using a plate reader (ALLSHENG, FlexA-200, China).

### Ferrous iron detection

FerroOrange (Dojindo, F374, China) was used to detect intracellular ferrous iron (Fe^2+^) according to the manufacturer’s protocol. In brief, RAW264.7 cells were infected by *T. gondii* RH-GFP tachyzoites for 24 h and stained with a final concentration of 1 µM FerroOrange for 30 minutes at 37°C. Subsequently, the cells were imaged using an inverted fluorescence microscope (Zeiss, Germany). Fe^2+^ fluorescence intensity was quantified using Image J software.

### Assessment of cytosolic ROS and lipid peroxidation

Cells were stained with 10 µM DCFH-DA or 2 µM BODIPY 581/591 C11 for 30 mins in the dark at 37°C. Cells were washed with phosphate-buffered saline (PBS), and then resuspended in 500 μL of fresh PBS, strained through a 40 μm cell strainer, and analysed using a flow cytometer (FACSuite, BD Biosciences, USA) equipped with a 488 nm laser for excitation.

### Quantitative real-time PCR

Total RNA was extracted from the cells and mice tissues using a Trizol reagent, as described by the manufacturer’s instructions (Invitrogen, USA). The integrity, quantity, and purity of RNA were examined using NanoDrop 2000c Spectrophotometer (Thermo Scientific, Wilmington, USA). The first-strand complementary DNA (cDNA) synthesis was performed using HiScript III RT SuperMix for qPCR (Vazyme, R323-01, China) according to the manufacturer’s instructions. Quantitative real-time (qRT)-PCR reactions were performed using the Taq Pro Universal SYBR qPCR Master Mix (Vazyme, Q712-02, China) following the manufacturer’s instructions. The internal gene mouse *β-Actin* or homo sapiens *GAPDH* was used as a reference to normalize the gene expression data. The relative gene expression was calculated using the 2^−ΔΔCt^ method. The sequences of the primers used for the qRT-PCR are listed in Table S2.

### Western blot analysis

The cell pellets were lysed in radioimmunoprecipitation assay (RIPA) buffer (Beyotime, P0013B, China) supplemented with protease inhibitor cocktail (APEXBIO, K1007, China) on ice for 30 min and centrifuged at 16,000 × g for 30 min to obtain the total cellular protein. Protein concentration was quantified in the supernatant using a BCA Protein Assay Kit (Beyotime, P0012, China). The proteins of interest were detected by immunodetection with the appropriate antibodies and chemiluminescence. Briefly, protein samples (10–30 μg) were separated by using 8–12% gradient sodium dodecyl sulphate-polyacrylamide gel electrophoresis (SDS-PAGE) gels and subsequently transferred into a 0.45 µm polyvinylidene difluoride (PVDF) membrane (Millipore, ISEQ00010, USA) The membranes were blocked with 5% skim milk in TBS containing 0.1% Tween (TBST) at room temperature for 2 h and incubated with incubated with specific primary antibodies overnight at 4 °C. After washing the membranes three times with 1×TBST, the membranes were incubated with horseradish peroxidase (HRP)-conjugated secondary antibodies for 1 h at room temperature. After 1.5 h washing, protein bands were visualized using chemiluminescent HRP substrate (Millipore, WBKLS0500, USA). Protein bands were visualized and the levels of protein expression were measured using a fully automated chemiluminescence image analysis system (Tanon, 4600SF, China).

### RNA-Seq analysis

Tachyzoites of *T. gondii* were treated with 0.1% DMSO or RSL3(5 µM) for 9 h. Untreated parasites were used as control group (Ctrl). Total RNA was extracted and purified using TRIzol Reagent (Invitrogen, USA) Transcriptome sequencing was performed as previously described [23]. Total RNA was fragmented and enriched using oligo (dT) magnetic beads, followed by cDNA synthesis. Sequencing libraries were generated after the removal of ribosomal RNA. Libraries were sequenced using DNBSEQ-500 platform, averagely generated more than 6 Gb bases per sample. Low quality reads and reads with adaptors or with unknown bases (N bases more than 5%) were filtered to get the clean read. After filtering, the clean reads with high quality were aligned to the *T. gondii* reference genome (https://www.ncbi.nlm.nih.gov/genome/30?genome_assembly_id = 899143) using HISAT2 (v2.0.4). Finally, clean reads were normalized to FPKM (fragments per kilobase of exon model per million mapped fragments) as relative gene expression levels. Differential gene expression analysis was performed using the DESeq2 Bioconductor package (v3.16), and the Benjamini–Hochberg multiple correction test was applied to control false discovery rate (FDR). Differentially expressed (DE) genes with an adjusted *p* value cut-off of 0.05 were considered statistically significant for further analysis. Gene ontology (GO) enrichment analysis of DE mRNAs was done using web-based GO software (http://www.geneontology.org). GO terms with a corrected *p* value (*Q* value) less than 0.05 were considered significantly enriched. Pathway enrichment analysis was performed by using a web-based Kyoto Encyclopedia of Genes and Genomes database (KEGG, http://www.genome.p/kegg)

### Parasite load determination

Parasite burden was measured using absolute quantitative PCR as previously described [23]. Samples for testing were carefully collected, and genomic DNA (gDNA) was extracted from them following the detailed operating instructions of the TIANamp Genomic DNA kit (DP304, TIANGEN, China). After extraction, the concentration and purity of the gDNA were accurately determined using a Nanodrop 2000 spectrophotometer (Thermo Scientific) to assess its quality for subsequent molecular analyses. To specifically and efficiently amplify the B1 gene of *T. gondii*, a pair of highly specific primers were meticulously designed. The forward primer sequence was 5”-TGAGTATCTGTGCAACTTTGG-3,‘ and the reverse primer sequence was 5’-TCTCTGTGTACCTCTTCTCG-3,” designed based on the known sequence of the *T. gondii* B1 gene to ensure high specificity and sensitivity in the PCR amplification process. A plasmid containing the B1 gene was used to prepare a series of standard samples with known concentrations; the original plasmid was serially diluted 10 - fold to obtain standard samples with concentrations of 10^1^, 10^2^, 10^3^, 10^4^, 10^5^, 10^6^, and 10^7^ copies/μL, with three replicates for each dilution to ensure the accuracy and reproducibility of the standard curve. FastKing One Step RT-PCR MasterMix (TIANGEN, China) were used for the real-time quantitative PCR reaction. After the reaction, the data were analysed using the CFX Manager software (provided by BIO-RAD). Based on the Ct values from the standard curve and the known copy numbers of the standard samples, a linear regression equation was established to calculate the copy number of the B1 gene of *T. gondii* in each sample, providing accurate and reliable quantification results.

### Mice infection assays

For ferroptosis-related factor detection, mice were randomly divided into a non-infected (control) group and an infected group (*n* = 5 per group). Mice in infected group were intraperitoneally (i.p.) injected with *T. gondii* RH tachyzoites (100 per mouse) in 100 µL PBS, while mice in the control group received the same volume of PBS as the infected group, but no parasite was added. On day 8 post-infection, all the animals were humanely euthanized, and the liver, kidney and spleen tissues were collected. RNA and protein were extracted and used for qRT-PCR and Western blotting analysis.

To evaluate the effect of RSL3 on *T. gondii* infection, mice were randomly divided into four groups (*n* = 15 per group), including vehicle group (6.7% DMSO, 40% PEG-300, 10% Tween 80, and ddH_2_O, i.p.), RSL3-pre group (50 mg/kg in 6.7% DMSO, 40% PEG-300, 10% Tween 80, and ddH_2_O, i.p.), RSL3-post group, and mock group. Schematic illustration of the infection and treatment protocol is shown in Figure 6a. Mice (*n* = 5) from each group were euthanized 9 days after *T. gondii* infection to obtain organ tissues for assessment of the parasite burden and histopathological analysis.

### Histopathological analysis

The spleen was fixed in 10% buffered formalin, dehydrated, and embedded in paraffin. Sections were cut at 5 μm, stained with haematoxylin-eosin, and examined under 100 × magnification using an inverted microscope (Zeiss, Germany).

### Statistical analysis

All statistical analyses were performed using Prism 8.0 (GraphPad Software, USA). All data are presented as mean ± SD based on at least three independent experiments. Statistical analyses were performed using unpaired two-tailed *t-*test to compare differences between two groups or one-way analysis of variance (ANOVA) when comparing three or more groups. A log-rank (Mantel-Cox) test was used for the mouse survival study. Statistical differences were considered statistically significant when *p* < 0.05, with asterisks denoting the degree of significance as follows: *, *p* < 0.05; **, *p* < 0.01; ***, *p* < 0.001, ****, *p* < 0.0001.

## Results

### T. gondii infection induces ferroptosis in macrophages and murine tissues

**Figure 1.**
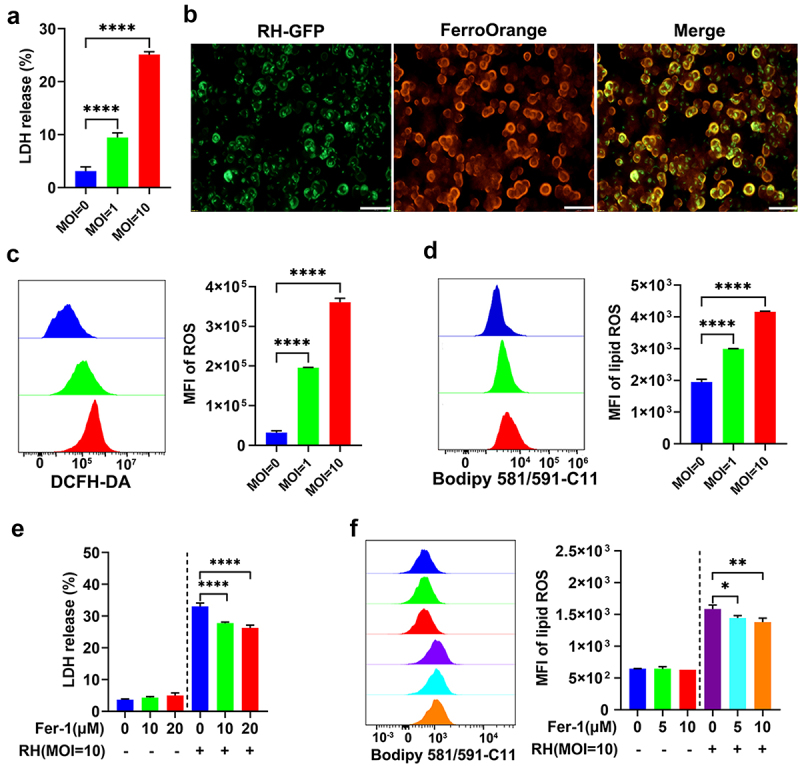
*T. gondii* infection triggers ferroptosis in macrophages. (a) A significant increase in lactate dehydrogenase (LDH) release was detected in RAW264.7 cells infected by *T. gondii* in an infection dose-dependent manner. (b) RAW264.7 cells treated with 1 μM FerroOrange revealed increased intracellular Fe^2+^ accumulation 24 h post-infection. Scale bars = 100 μm. (c) An elevated production of the reactive oxygen species was detected in *T. gondii*-infected RAW264.7 cells using the DCFH – DA assay. (d) Increased lipid peroxidation was detected in infected cells through the application of a BODIPY 581/591 C11 fluorescent probe. Treatment with Fer-1 decreased the levels of LDH (e) and lipid ROS (f) in *T. gondii*-infected RAW264.7 cells in a dose-dependent manner. Cells were pre-incubated with varying concentrations of Fer-1 prior to *T. gondii* infection. Two hours later, cells were infected with *T. gondii* tachyzoites (MOI = 10), and supernatants were collected 24 h post-infection for LDH measurement and lipid ROS detection. The data are shown as the mean ± SD of at least three independent experiments. Statistical significance was calculated using Student’s *t* test for two groups and one-way ANOVA for more than two groups followed by Tukey’s multiple comparison test. *p < 0.05, **p < 0.01, ***p < 0.001, ****p < 0.0001, compared with the control group.

To test whether infection by *T. gondii* elicits ferroptosis in host cells, RAW264.7 macrophages were infected with *T. gondii* RH tachyzoites at different MOIs (0, 1, 10) for 24 h. As shown in Figure 1a, infection reduced cell viability and increased lactate dehydrogenase (LDH) release in a MOI-dependent manner, indicative of plasma membrane damage. Intracellular labile ferrous ion (Fe^2+^) accumulation, a hallmark of ferroptotic stress, was markedly elevated in infected cells (Figure 1b), with significant increases relative to uninfected controls confirmed by quantitative analysis (Figure S1). Infection also induced cytosolic reactive oxygen species (ROS) and lipid peroxidation, as measured by DCFH-DA and BODIPY 581/591 C11 staining, respectively (Figure 1c,d). Given that redox-active iron accumulation and lipid peroxidation are central to ferroptosis, we assessed whether ferroptosis inhibition could ameliorate infection-induced cytotoxicity. Pre-treatment with the ferroptosis-specific inhibitor Fer-1 significantly attenuated LDH release (Figure 1e) and mitigated *T. gondii*-induced BODIPY C11 oxidation (Figure 1f), indicating suppression of lipid peroxidation. These results suggest that *T. gondii* infection triggers ferroptotic cell death in host macrophages, characterized by iron overload, ROS generation, and lipid peroxidation, which can be pharmacologically modulated by ferroptosis inhibitors.

**Figure 2.**
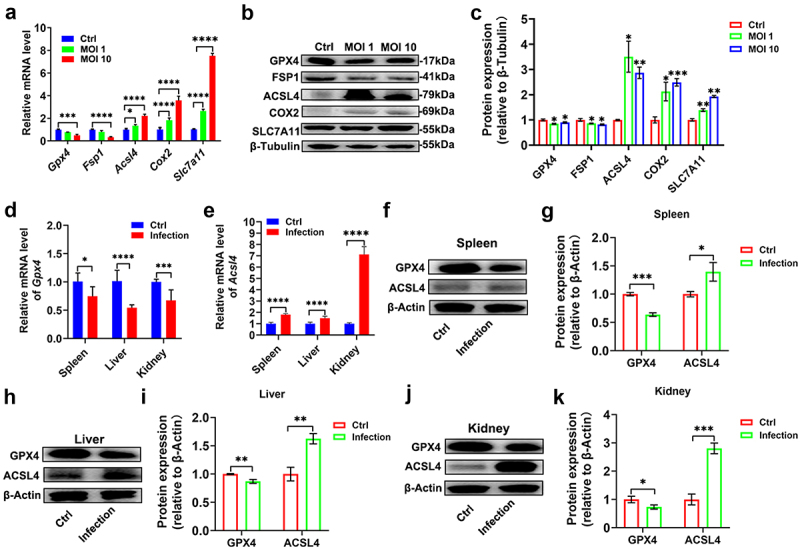
*T. gondii* infection modulates the expression of ferroptosis-related factors in vitro and in vivo. (a) mRNA expression levels of indicated ferroptosis-related genes in RAW264.7 cells at 24 h after *T. gondii* infection (MOI 1 or 10) relative to uninfected controls. (b) Western blot analysis of ferroptosis-related proteins in RAW264.7 cells at 24 h after infection. Primary antibodies against GPX4 (1:1000), FSP1 (1:1000), ACSL4 (1:10,000), COX2 (1:1000), SLC7A11 (1:1000), and β-tubulin (1:10,000) were used, followed by goat anti-rabbit HRP-conjugated secondary antibody (1:10,000). (c) Densitometric quantification of Western blot signals from (B), normalized to β-tubulin. (d,e) qPCR analysis of GPX4 and ACSL4 mRNA expression in spleen, liver, and kidney tissues from infected mice. (f,h,j) Western blot analysis of GPX4 and ACSL4 protein levels in murine splenic (f), hepatic (h), and renal (j) tissues. Primary antibodies: GPX4 (1:1000), ACSL4 (1:10,000), β-actin (1:10,000); secondary antibody: goat anti-rabbit HRP-conjugated (1:10,000). (g,i,k) quantification of Western blot signals from (f,h,j), normalized to β-actin. All data represent the mean ± SD from at least three independent experiments. Statistical significance was determined using Student’s *t*-tests for pairwise group comparisons or two-way ANOVA with Tukey’s post-hoc multiple comparison test for multi-group analyses. *p < 0.05, **p < 0.01, ***p < 0.001, ****p < 0.0001, compared to the control.

Ferroptosis-associated gene expression profiles, including SLC7A11, ACSL4,FSP1, COX2, and GPX4, were analysed in *T. gondii*-infected RAW264.7 cells via qRT-PCR and Western blotting. At both the transcriptional and translational levels, *T. gondii* infection (MOI 1 or 10, 24 h post-infection) significantly upregulated SLC7A11, ACSL4, and COX2 compared to uninfected controls (Figure 2a–c). Conversely, GPX4 and FSP1 mRNA and protein levels were markedly reduced in infected cells. To validate these findings in vivo, we assessed GPX4 and ACSL4 expression in the spleen, liver, and kidney of infected mice using qRT-PCR and Western blot analysis. Consistent with in vitro results, infected tissues exhibited decreased GPX4 and increased ACSL4 expression at both the mRNA and protein levels (Figure 2d–k). These coordinated changes in key ferroptosis regulators-upregulation of pro-ferroptotic factors and downregulation of the ferroptosis suppressor GPX4-indicate that *T. gondii* infection modulates the host ferroptosis pathway both in vitro and in vivo, potentially facilitating an iron-lipid metabolic environment conducive to parasite survival.

#### SLC7A11/GPX4 signalling pathway plays a crucial role in the proliferation of T. gondii

**Figure 3.**
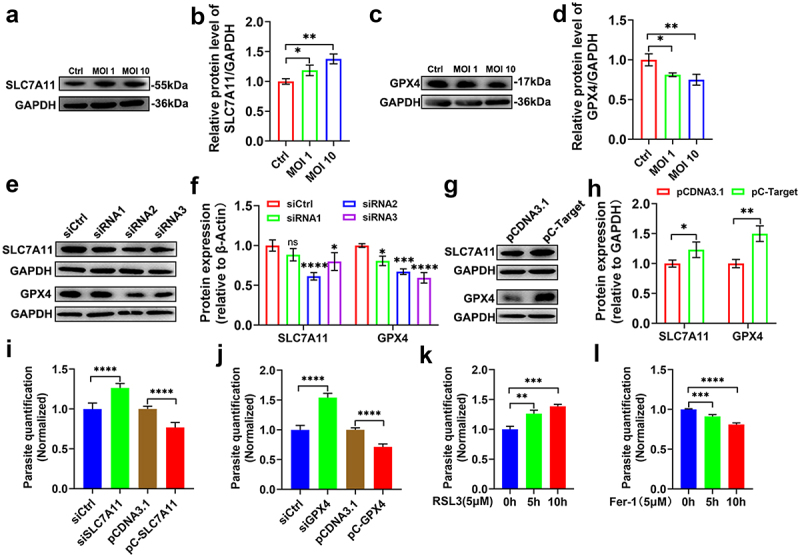
Inhibition of the SLC7A11/GPX4 signalling pathway promotes *T. gondii* proliferation. (a,c) HeLa cells were infected with *T. gondii* at an MOI 0, 1, or 10 for 24 h. Total protein lysates were analysed by Western blotting using primary antibodies against SLC7A11 (1:1000), GPX4 (1:1000) and GAPDH (1:10,000 dilution), followed by goat anti-rabbit HRP-conjugated secondary antibody (1:10,000). (b,d) Densitometric quantification of Western blot signals from (a,c), normalized to GAPDH. (e,g) Knockdown or over-expression efficiency of SLC7A11/GPX4 was validated by Western blotting using the same antibody panel as in (a,c). siRNA2 for SLC7A11 and siRNA3 for GPX4 exhibited high knockdown efficiency. (i) Knockdown of SLC7A11 in HeLa cells increased parasite proliferation, whereas transient overexpression of SLC7A11 reduced proliferation. At 48 h after siRNA or overexpression plasmid transfection, HeLa cells were infected with *T. gondii* at MOI 1. Cells were collected 48 h post-infection to measure intracellular parasite load. (j) Knockdown of GPX4 increased parasite proliferation, while transient overexpression of GPX4 suppressed proliferation. (k) Pre-treatment with the ferroptosis inducer RSL3 prior to infection promoted parasite proliferation. (l) Pre-treatment with the ferroptosis inhibitor Fer-1 prior to infection inhibited parasite proliferation. All data are shown as the mean ± SD from at least three independent experiments. Statistical analysis by two-sided one-way ANOVA corrected for multiple comparisons using Tukey’s multiple comparison test or by parametric *t*-test. *p  < 0.05, **p < 0.01, ***p < 0.001, ****p < 0.0001.

To precisely assess the impact of SLC7A11/GPX4 signalling axis on the growth dynamics of *T. gondii*, we conducted a series of experiments. Specifically, we measured the intracellular replication of the parasite within HeLa cells, which have become a commonly used model for transfection experiments. We manipulated the expression levels of SLC7A11/GPX4 by either depleting these proteins through siRNA treatment or overexpressing them. In comparison to uninfected cells, the protein level of SLC7A11 was upregulated by *T. gondii* in a manner that was dependent on MOI (Figure 3a,b). Conversely, the infection led to a decrease in the protein level of GPX4 (Figure 3c,d). Next, we transfected a set of candidate siRNA molecules or overexpression plasmids into HeLa cells and allowed them to incubate for 48 h. We then determined the protein levels of SLC7A11/GPX4 via Western blot analysis (Figure 3e–h). For the subsequent experiments, we selected siRNA2 targeting SLC7A11 and siRNA3 targeting GPX4, as these two siRNAs demonstrated an inhibition efficiency of approximately 40%. Next, we employed quantitative real-time polymerase-chain reaction (qPCR) to quantify the effects of knocking down and overexpressing SLC7A11/GPX4 on the growth of the parasite. Our findings revealed that the knockdown of SLC7A11/GPX4 facilitated the proliferation of the parasite, while the high expression of SLC7A11/GPX4 reduced parasite proliferation (Figure 3i,j). Furthermore, we treated RAW264.7 cells with RSL3, an inducer of ferroptosis, or the ferroptosis inhibitor Fer-1 (at a concentration of 5 μM) for 5 h or 10 h, followed by infection with *T. gondii* tachyzoites at a MOI of 1. As shown in Figure 3k, pretreatment with RSL3 significantly enhanced parasite proliferation. In contrast, the pretreatment of cells with Fer-1 led to a significant inhibition of parasite proliferation, as shown in Figure 3l. Collectively, these results unequivocally demonstrate the pivotal role of the SLC7A11/GPX4 signalling pathway during *T. gondii* infection.

#### Inhibitory effects of RSL3 against T. gondii tachyzoites in vitro

**Figure 4.**
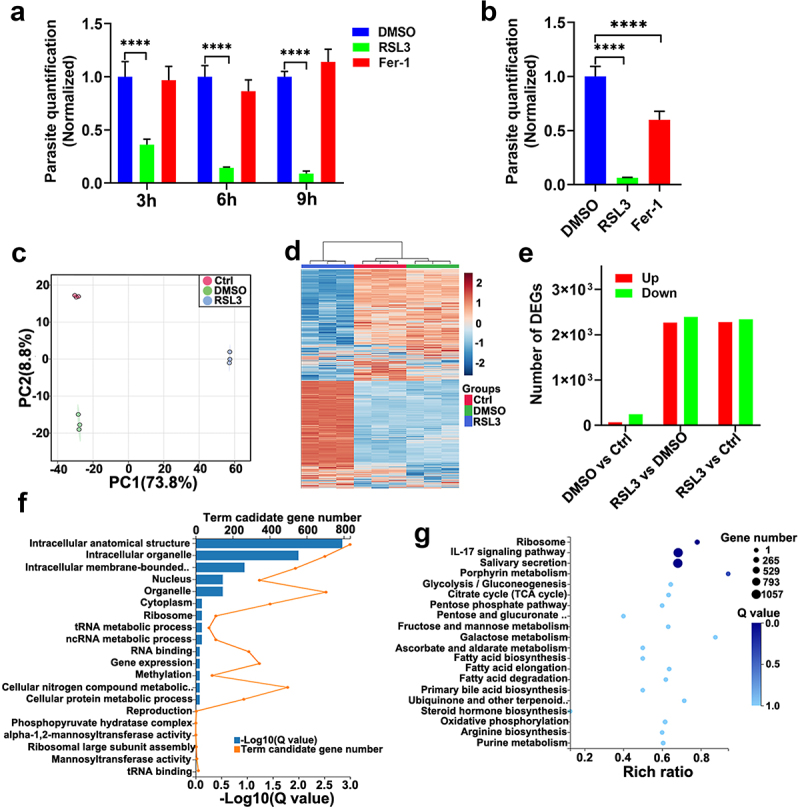
RSL3 has an anti-*T. gondii* effect and alters the transcriptome of *T. gondii* RH tachyzoites. (a) Effects of RSL3 or Fer-1 (5 μM) on the activity of extracellular *T. gondii* tachyzoites. Tachyzoites were incubated with 0.1%DMSO, 5 µM RSL3, or 5 µM Fer-1 for 3, 6, or 9 hours, then washed and used to infect RAW264.7 macrophages at an MOI of 1. After 48 hours, cells were harvested to quantify the intracellular parasite burden. *p* values were calculated by two-way ANOVA; ****p < 0.001. (b) Inhibitory effects of RSL3 or Fer-1 (5 μM) on intracellular parasite replication. RAW264.7 cells were infected with *T. gondii* tachyzoites at an MOI of 1 for 2 hours, washed to remove non-internalized parasites, and then incubated with 0.1% DMSO, 5 µM RSL3 or 5 µM Fer-1 for 48 hours. Cells were collected to evaluate the parasite burden. *p* values were calculated by one-way ANOVA; ****p < 0.001. (c) Principal component analysis (PCA) scatter plot of *T. gondii* transcriptomes following treatment with the indicated compounds, based on fragments per kilobase of transcript per million mapped reads (FPKM) values. (d) Heatmap depicting differentially expressed genes (DEGs) across the indicated sample groups, clustered by expression profiles. (e) Summary of upregulated and downregulated genes in pairwise comparisons between treatment groups. (f) Gene ontology (GO) enrichment analysis of DEGs. The x-axis represents −log₁₀(Q-value) (adjusted *p*-value), and the y-axis lists enriched GO terms. (g) Top 20 significantly enriched Kyoto Encyclopedia of genes and Genomes (KEGG) pathways of DEGs, ranked by rich ratio (proportion of DEGs in the pathway relative to all genes in the reference genome). The x-axis denotes the rich ratio, and the y-axis lists the KEGG pathways.

To further investigate whether ferroptosis regulators (inducers and inhibitors) have a direct inhibitory effect on *T. gondii* tachyzoites, we first evaluated the efficacy of RSL3 or Fer-1 on extracellular tachyzoites. The extracellular parasites exposed to RSL3 exhibited growth impairment in a time-dependent manner, whereas Fer-1 had no significant effect on the parasite proliferation (Figure 4a). For intracellular parasites, we found that both RSL3 and Fer-1 showed significant inhibitory effects, but the effect was more prominent with RSL3 (Figure 4b). Next, we analysed the effect of RSL3 on *T. gondii* transcriptome. The Principal Components Analysis (PCA) scores plot and unsupervised hierarchical clustering clearly demonstrated a distinct separation among parasites treated with RSL3, those treated with the vehicle (0.1% DMSO) and the negative control (Ctrl) (Figure 4c,d). Between RSL3-treated parasites and 0.1% DMSO-treated parasites, 2,280 genes were identified as up-regulated, and 2,342 genes were down-regulated, as shown in Figure 4e. Detailed information regarding these differentially expressed genes (DEGs) is provided in Table S3. To gain a better understanding of the biological function of these DEGs, Gene Ontology (GO) enrichment and Kyoto Encyclopedia of Genes and Genomes (KEGG) pathway analysis were performed. As demonstrated in Figure 4f, the three most significantly enriched GO terms were “intracellular anatomical structure,” “intracellular organelle,” and “intracellular membrane-bounded organelle.” Moreover, as shown in Figure 4g, the “Ribosome” pathway was the most affected among all the pathways analysed.

#### Ferroptosis regulators impact the antioxidant capacity of T. gondii

**Figure 5.**
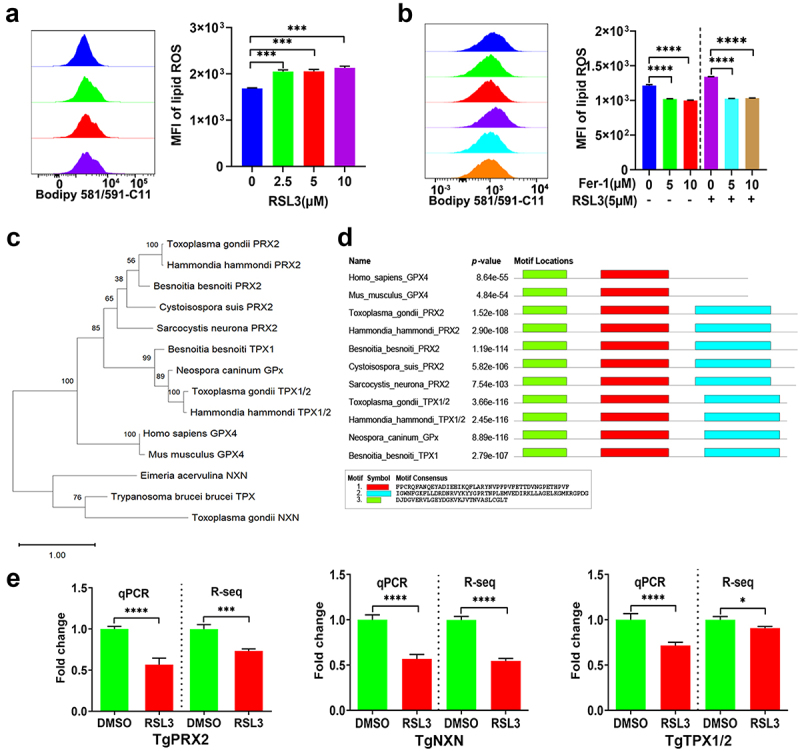
RSL3 induces lipid ROS accumulation and suppresses antioxidant gene expression in *T. gondii*. (a) RSL3 treatment induces dose-dependent accumulation of lipid ROS in *T. gondii* tachyzoites after 9 h of incubation. (b) Fer-1 attenuates lipid ROS production in *T. gondii*, even in the presence of the inducer RSL3. (c) Phylogenetic tree of GPX4 and Tpx homologs across divergent species, constructed using amino acid sequences. (d) Conserved putative motifs in GPX4 homologous proteins identified by MEME analysis. Distinct colored rectangles represent unique conserved motifs, with protein and motif lengths scalable via the provided bar. (e) qPCR and RNA-seq analysis of TgPRX2, TgTPX1/2, and TgNXN expression in *T. gondii* RH strain treated with vehicle (0.1% DMSO) or RSL3 (5 µM) for 9 h. Two-group comparisons were analyzed using Student’s *t* test, while multi-group comparisons employed one-way ANOVA with Tukey’s post-hoc multiple comparison test.

To explore whether ferroptosis regulators affect the antioxidant activity of *T. gondii*, we measured the levels of lipid ROS of parasite after treatment with RSL3 or Fer-1. As shown in Figure 5a, RSL3 treatment significantly increased the lipid ROS levels in extracellular tachyzoites (*p* < 0.001). Conversely, treating tachyzoites with varying concentrations of Fer-1 led to a reduction in lipid ROS production and counteracted the stimulatory effect of RSL3 on lipid ROS generation (Figure 5b). Tryparedoxin (Tpx) is the closest orthologue of mammalian GPX4 and plays a pivotal role in redox-metabolism in trypanosomes. This protein can be inhibited by the ferroptosis inducer RSL3 [24]. We performed a BLAST search in the sequence databases for *T. gondii* orthologs of GPX4 and Tpx. As shown in Figure 5c, TgPRX2 (TGGT1_266130) and TgTPX1/2 (TGGT1_266120) were found to be homologous to GPX4, while TgNXN (TGGT1_225060) was homologous to Tpx. Conserved motif domains were identified by aligning the protein sequences using the MEME program. Two main motifs were present in TgPRX2, TgTPX1/2 and GPX4 (Figure 5d). The mRNA expression levels of TgPRX2, TgTPX1/2 and TgNXN, determined by qPCR and RNA-Seq, were consistent (Figure 5e). These levels decreased after RSL3 treatment, indicating a compromised ability of the parasite to manage oxidative damage.

#### RSL3-induced ferroptosis facilitates T. gondii infection in vivo

**Figure 6.**
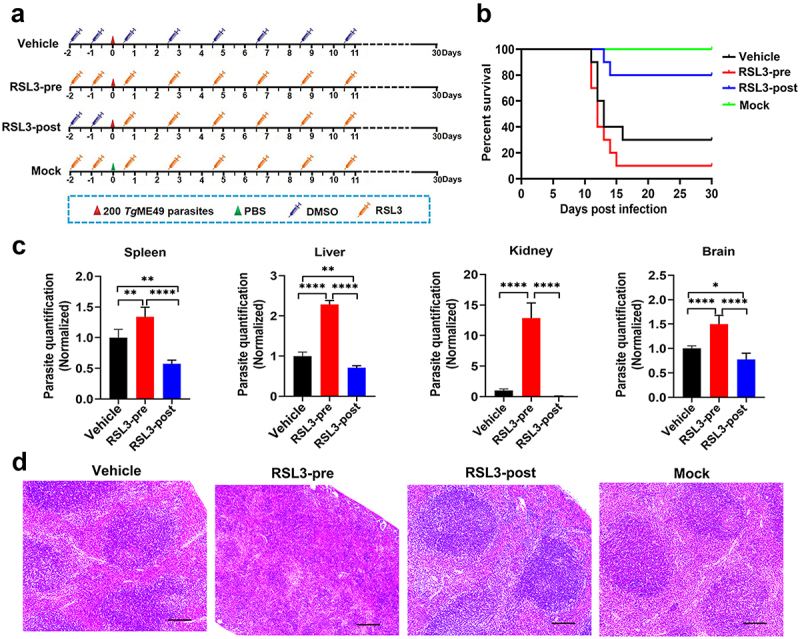
Pre-infection treatment with the ferroptosis inducer RSL3 enhances susceptibility to *T. gondii* infection in mice. (a) Schematic of the experimental design for *T. gondii* infection and RSL3 treatment (*n* = 10 mice per group). RSL3 was administered intraperitoneally (i.p., 50 mg/kg) either 48 h before infection (RSL3-pre) or 12 h after infection (RSL3-post). (b) Survival curve analysis showed that post-infection RSL3 treatment improved host survival, whereas pre-infection RSL3 administration exacerbated infection severity. (c) Parasite burden in spleen, liver, kidney, and brain tissues was quantified by qPCR, normalized to vehicle group. Pre-infection RSL3 treatment significantly increased parasite load, while post-infection treatment reduced it relative to the vehicle control. Data are analyzed with one-way ANOVA followed by Tukey’s multiple comparison test. (d) Representative H&E-stained histological sections of splenic tissues from each group at 9 days post-infection with the *T. gondii* ME49 strain. Mice pre-treated with RSL3 exhibited significant structural alterations in splenic architecture compared to controls. Scale bars = 100 μm.

To evaluate the effects of RSL3 on the progression of infection within a mammalian host, we designed experiments as outlined in Figure 6a. When compared with the vehicle control group (receiving solvent alone) and PBS-treated negative control group, post-infection administration of RSL3 delayed the appearance of clinical symptoms and improved survival outcomes in mice (Figure 6b). Conversely, pre-infection treatment with RSL3 resulted in a decreased survival rate, indicating that RSL3-mediated ferroptosis may facilitate parasite proliferation and accelerate infection progression in vivo. To validate this finding, we quantified parasite burdens in the spleen, liver, kidney, and brain of mice from four experimental groups-vehicle control, PBS control, pre-infection RSL3 treatment (RSL3-pre), and post-infection RSL3 treatment (RSL3-post)-at 9 days post-infection. This humane endpoint time was chosen based on the time at which mice in the most vulnerable group (RSL3-treated before infection group) began showing clinical signs indicative of toxoplasmosis before the onset of distress. Notably, the RSL3-pre group exhibited significantly higher parasite loads in all analysed organs compared to other groups (Figure 6c). Histopathological analysis of splenic tissues revealed that pre-infection RSL3 treatment was associated with splenomegaly, characterized by reduced white pulp architecture and expanded red pulp compartments, indicative of immune microenvironment dysregulation induced by *T. gondii* (Figure 6d). In contrast, administration of the ferroptosis inhibitor Fer-1—either pre- or post-infection – had no observable effect on mouse survival rates (data not shown). In conclusion, RSL3-induced ferroptosis promoted the replication of *T. gondii* in vivo, and this promoting effect overshadowed the potential anti-*T. gondii* effect of RSL3.

## Discussion

Emerging evidence indicates that ferroptosis is implicated in the pathogenesis of several infectious diseases, indicating that targeting ferroptosis may offer a novel therapeutic approach to control these diseases. However, the role of ferroptosis in *T. gondii* infection is poorly understood. In this study, we show that *T. gondii* can induce ferroptosis in RAW264.7 cells and in mice. Inhibition of SLC7A11/GPX4 signalling pathway induced ferroptosis and increased parasite replication. *T. gondii* replication was also significantly increased in RAW264.7 cells and mice pretreated with RSL3, suggesting that ferroptosis may accelerate infection progression. Paradoxically, ferroptosis inducer RSL3 increased lipid peroxidation and inhibited the expression of antioxidant genes in *T. gondii*, resulting in potent anti-*T. gondii* effects *in vitro* and *in vivo*.

Iron plays a key role in host–pathogen interaction, and accumulation of lipid-free radicals due to iron overload and dysregulation of antioxidative responses culminates in ferroptosis [9]. The dysregulation of iron homoeostasis and excess ROS production have been reported in several infections, such as *Pseudomonas aeruginosa*, *Mtb* and SARS-CoV-2 [25,26]. Latent infection by hepatitis C virus (HCV) is associated with elevated iron levels. Excess iron inhibits HCV replication by inhibiting the enzymatic activity of the RNA polymerase NS5B [27]. Treatment with deferoxamine mesylate (DFO) significantly limits hPIV-2 or HIV replication [28,29]. On the other hand, iron overload favours the growth of mycobacteria and accelerates the development of tuberculosis *in vivo* [30,31]. C57BL/6 mice infected by *T. gondii* exhibit increase in iron level and a reduction in parasite burden in the hippocampus following treatment with an iron-chelating agent deferiprone (DFP) [8]. Another iron-chelating agent, DFO, decreases *T. gondii* multiplication in human villous (BeWo) and extravillous (HTR-8/SVneo) trophoblast cells [32]. In the present study, *T. gondii* infection induced cell death and ROS production, and promoted intracellular Fe^2+^ in RAW264.7 cells. Additionally, a ferroptosis inhibitor Fer-1 ameliorated *T. gondii* infection-induced lipid peroxidation and downregulated LDH production. These findings suggest that RSL3-induced GPX4 deficiency increased intracellular lipid peroxides, which led to ferroptosis of RAW264.7 cells, thus weakening immunological response against *T. gondii* infection, allowing the parasites to proliferate. However, Fer-1 which inhibits lipid peroxidation has maintained the cellular redox homoeostasis of RAW264.7 cells and thus enhanced their anti-*T. gondii* response.

The findings from the present and previous studies showed that *T. gondii* growth depends on iron storage in host cells and that increase in the levels of lipid peroxidation results in ferroptotic cell death. Acsl4 is an essential pro-ferroptotic gene and an essential component in the ferroptosis process. Up-regulation of ACSL4 induced by iron‐uptake is required for GPX4 degradation [33]. In our study, we demonstrated this crucial role of GPX4 and ACSL4 as mediators of ferroptotic cell death, in the context of *T. gondii* infection. Decreased GPX4 expression and elevated the ACSL4 levels were detected in RAW264.7 cells infected by *T. gondii*. Acute *T. gondii* infection causes histopathology in the liver, spleen, and kidney [34–36]. In this study, we found that *T. gondii*-induced tissue pathology is associated with reduced GPX4 expression and high expression of ACSL4, suggesting a key role of GPX4 and ACSL4 in mediating the pathogenesis of toxoplasmosis.

The SLC7A11-GPX4 signalling pathway is one of the most classic ferroptosis defence pathways. Some pathogens exploit ferroptosis, by altering the expression of GPX4 and SLC7A11, to facilitate their replication and spread. For example, HIV promotes ferroptosis by suppressing GPX4 in T cells during the early stages of infection, which is critical for HIV pathogenesis [37]. *Leishmania* infection in GPX4-deficient mice leads to a reduction in the number of CD4^+^ T cells, which contributes to the establishment of infection [38]. Infection by Coxsackievirus A6 (CV-A6) induces ferroptosis *via* ACSL4 to facilitate its replication in host cells. Fer-1 inhibits the development of viral replication and lipid ROS levels, thus limiting CV-A6 replication [39]. In our study, proliferation of intracellular *T. gondii* was inhibited by overexpressing SLC7A11 or GPX4, whereas downregulation of SLC7A11 or GPX4 increased the parasite replication. In addition, parasite replication was significantly promoted in cells pretreated with RSL3, however parasite replication was significantly reduced in host cells treated with Fer-1. These results suggest that ferroptosis plays a role in the pathogenicity of *T. gondii* and that ferroptosis impairs host resistance to *T. gondii* infection.

The modulation of intracellular ROS levels is crucial for cellular homoeostasis and determination of cellular fate. Evidence shows that excessive accumulation of intracellular ROS in *T. gondii* leads to parasite damage [40]. In the present study, RSL3 exhibited a potent inhibitory effect on both intracellular and extracellular tachyzoites *in vitro*, which is consistent with a previous study [22]. RSL3 boosted lipid ROS levels in *T. gondii*. Also, it caused marked alterations in the parasite transcriptome and affected expression of genes associated with antioxidant activity and cell death. qPCR analysis revealed that the expression of antioxidant genes, such as TgPRX2, TgTPX1/2 and TgNXN were significantly suppressed, which is consistent with the RNA-Seq data. It is worth noting that Fer-1 showed no inhibitory effect on extracellular parasites, but had significant inhibitory effect on intracellular parasites, suggesting that Fer-1 inhibited ferroptosis caused by *T. gondii* and thus limited parasite growth.

In the mouse study, we found that RSL3 used pre-infection, via induction of ferroptosis, increased parasite replication and dissemination, and decreased the animal survival rate. However, RSL3 administered after infection significantly increased the survival rate of mice, while simultaneously lessened splenic tissue damage as well as decreased parasite burden in different organs. However, Fer-1 used pre- or post-infection did not ameliorate the tissue pathology or enhanced the survival of mice. A plausible explanation for the phenotype difference between *in vivo* and *in vitro* effect of Fer-1 treatment is that other systems may bypass the effect of Fer-1 on lipid peroxidation *in vivo*. Taken together, these results suggest that RSL3, through two different mechanisms involving the SLC7A11-GPX4 axis, can both restrain and promote parasite infection.

The paradoxical effect of RSL3 on the progression of *T. gondii* infection may have a plausible explanation in a context-dependent manner. We have shown that *T. gondii* infection reduces the expression of GPX4, and consequently induces ferroptosis, which was associated with improved parasite replication. Likewise, deletion or reduction of GPX4 using RSL3-induced ferroptosis and promoted *T. gondii* infection. Thus, the enhanced host cell ferroptosis caused by RSL3-mediated reduction in GPX4 prior to infection is likely to be the reason for the increased parasite propagation. The inhibition of parasite growth when RSL3 was administered post-infection may result from the direct inhibitory effect of RSL3 on the parasite. RSL3 downregulated the expression of genes involved in the antioxidant activity, redox homoeostasis, and cell growth and death pathways in *T. gondii*, which decreases ROS scavenging and makes *T. gondii* more vulnerable to the deleterious effects of oxidative stress and ROS-dependent cell death [41,42]. Whether ferroptosis benefits the host or parasite remains to be further elucidated; both outcomes might be possible at different stages of the host–pathogen interaction.

In conclusion, our data revealed that *T. gondii* infection induces ferroptosis to promote parasite proliferation and SLC7A11/GPX4 signalling pathway is required for parasite growth. SLC7A11/GPX4 knockdown promoted parasite proliferation, but SLC7A11/GPX4 overexpression reduced parasite proliferation. Pre-treatment with ferroptosis inducer RSL3 increased the severity of toxoplasmosis in mice, whereas treatment with RSL3 post infection reduced the clinical signs and prolonged the survival of infected mice, suggesting that ferroptosis can limit and promote toxoplasmosis in a paradoxical, context-dependent manner, depending on other regulatory factors induced by infection. Understanding the precise roles of ferroptosis when induced prior to and after *T. gondii* infection may offer a new perspective for modulating resistance and mitigating the clinical severity of toxoplasmosis or for the development of novel therapeutics against *T. gondii* infection.

## Supplementary Material

Table S1.doc

Figure S1.tif

ARRIVE guidelines.pdf

Table S2.doc

## Data Availability

The transcriptome data that support the findings in Figure 4 are openly available in the ScienceDB (https://doi.org/10.57760/sciencedb.18764) and in the NCBI database at https://www.ncbi.nlm.nih.gov/bioproject/?term = PRJNA922691, reference number PRJNA922691.The detailed data for Table S3 are available in the figshare repository (https://doi.org/10.6084/m9.figshare.27950625.v2).
