# Supplementary material for: Ferroptosis is important for Toxoplasma gondii replication and virulence in vitro and in vivo
Source: Virulence. 2025 Jul 16;16(1):2530164. doi: 10.1080/21505594.2025.2530164 (PMC12269666; doi:10.1080/21505594.2025.2530164)
Supplement: Table S1.doc [file KVIR_A_2530164_SM7076.doc]

**Table S1 Primers used to construct plasmids in this study.**

| Primer name | Primer sequence (5’-3’) |
| --- | --- |
| *slc7a11* | Forward: CCCAAGCTTGCCACCATG GTCAGAAAGCCTGTTGTGTCC  Reverse: ATCGTCGTCCTTGTAGTCGAATTCTAACTTATCTTCTTCTGGTACAACTTC |
| *gpx4* | Forward: CCCAAGCTTGCCACCATG AGCCTCGGCCGCCTTT  Reverse: ATCGTCGTCCTTGTAGTCGAATTC TGAGTGCCGGTGGAAGGC |
| *pcDNA3.1-FLAG* | Forward: GAATTCGACTACAAGGACGACGAT |
|  | Reverse: CATGGTGGCAAGCTTGGG |
