# Supplementary figures and images for: Ferroptosis is important for Toxoplasma gondii replication and virulence in vitro and in vivo
Source: Virulence. 2025 Jul 16;16(1):2530164. doi: 10.1080/21505594.2025.2530164 (PMC12269666; doi:10.1080/21505594.2025.2530164)

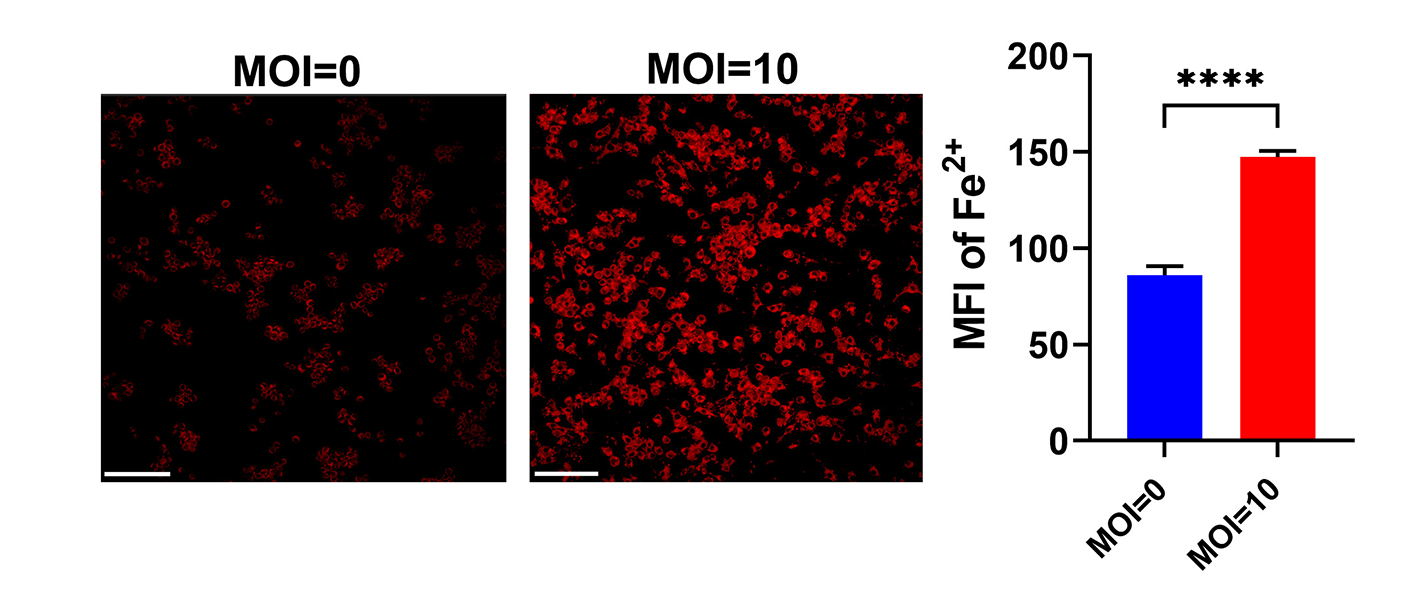

Supplement: Figure S1.tif [file KVIR_A_2530164_SM7075.tif]
