# Supplementary material for: Ferroptosis is important for Toxoplasma gondii replication and virulence in vitro and in vivo
Source: Virulence. 2025 Jul 16;16(1):2530164. doi: 10.1080/21505594.2025.2530164 (PMC12269666; doi:10.1080/21505594.2025.2530164)
Supplement: Table S2.doc [file KVIR_A_2530164_SM7073.doc]

**Table S2 Primers used for qPCR**

| Primer name | Primer sequence (5’-3’) |
| --- | --- |
| *Mus-β-actin* | Forward：5’ -GGCTGTATTCCCCTCCATCG-3’  Reverse：5’ -CCAGTTGGTAACAATGCCATGT-3’ |
| *Mus-gpx4* | Forward：5’ -GCAACCAGTTTGGGAGGCAGGAG-3’  Reverse：5’ -CCTCCATGGGACCATAGCGCTTC-3’ |
| *Mus-acsl4* | Forward：5’ -TGGCTACTTACCTTTGGCTCATGTG-3’  Reverse：5’ -ACAATCACCCTTGCTTCCCTTCTTG-3’ |
| *Mus-ptgs2* | Forward：5’ -TGCTGTACAAGCAGTGGCAA-3’  Reverse：5’ -CAGCCATTTCCTTCTCTCCTGT-3’ |
| *Mus-fsp1* | Forward：5’ -ACCGCAGTGCATTTGAGAGT-3’  Reverse：5’ -GGTATCGGCACAGTCACCAA-3’ |
| *Homo-slc7a11* | Forward：5’ -ATGCAGTGGCAGTGACCTTT-3’ |
|  | Reverse：5’ -CATGGAGCCAAAGCAGGAGA-3’ |
| *Homo-gpx4* | Forward：5’ -AAGATCCAACCCAAGGGCAA-3’ |
|  | Reverse：5’ -AGACGGTGTCCAAACTTGGTG-3’ |
| *Homo-gapdh* | Forward：5’ -GAAAGCCTGCCGGTGACTAA-3’ |
|  | Reverse：5’ -GCCCAATACGACCAAATCAGAG-3’ |
